# Biofeedback Fixation Training in the Rehabilitation of Patients with Geographic Atrophy

**DOI:** 10.3390/life16010165

**Published:** 2026-01-19

**Authors:** Kristóf Vörös, Illés Kovács, Gréta Kézdy, Ágnes Élő, Zsuzsa Szilágyi, Mirella Barboni, Zsuzsa Récsán, Zoltán Zsolt Nagy, Monika Ecsedy

**Affiliations:** Department of Ophthalmology, Semmelweis University, 1085 Budapest, Hungary

**Keywords:** biofeedback fixation training, geographic atrophy, rehabilitation

## Abstract

Geographic atrophy (GA) is a progressive cause of central vision loss with limited rehabilitation options. This prospective case series aimed to evaluate the effects of biofeedback fixation training (BFT) on visual function and vision-related quality of life (QoL) in patients with GA. Eighteen patients with total central vision loss in one eye underwent BFT on the fellow eye (study eye) using the Macular Integrity Assessment (MAIA) system, which was used to select a new, previously chosen preferred retinal locus (PRL) to stabilize fixation or adopt a new fixation locus. Patients were followed for an average of 13.2 months (range 3–26 months). Functional outcomes included best corrected visual acuity (ETDRS chart), reading performance (Radner test), and contrast sensitivity (Spot Checks test). MAIA parameters comprised average retinal sensitivity, fixation distance and stability (P1, P2), and changes in the bivariate contour ellipse area (BCEA). Vision-related quality of life was assessed using the National Eye Institute Visual Functioning Questionnaire-25 (NEI-VFQ-25). Following BFT, visual acuity, reading ability and contrast sensitivity improved significantly (*p* value: *p* < 0.02), and fixation stability and NEI-VFQ-25 scores showed a positive trend. These findings indicate that BFT is a feasible and promising rehabilitation approach for patients with GA.

## 1. Introduction

Age-related macular degeneration (AMD) is the leading cause of vision loss in developed countries, particularly among individuals older than 60 years, and its prevalence is expected to rise due to exponential population ageing [1]. It has also become more concerning in the developing countries as the patients present late [2]. The two major forms of the disease are neovascular (nAMD) and dry AMD [3]. In both types, the advanced stages are characterized by progressive and irreversible loss of central vision caused by damage to the retinal pigment epithelium, photoreceptors, and underlying choriocapillaris [3]. Substantial progress has been made in the management of nAMD with the introduction of anti-angiogenesis therapy, which has provided patients with effective treatment options that can prevent blindness and, in many cases, restore vision [4]. However, no such advances exist for the approximately 6 million individuals worldwide who are estimated to have late-stage dry AMD, commonly termed geographic atrophy (GA), which can also be loaded by the long-term use of anti-angiogenetic agents in nAMD patients. Two complement system inhibitors—pegcetacoplan, a C3 inhibitor (Syfovre; Apellis Pharmaceuticals Inc., Waltham, MA, USA), and avacincaptad pegol, a C5 inhibitor (Izervay; Astellas Pharma Inc., Tokyo, Japan)—have been approved by the U.S. Food and Drug Administration and have demonstrated the ability to slow GA progression. Nevertheless, neither therapy is capable of improving visual function, and in Europe, the European Medicines Agency recently rescinded its market approval [5,6,7,8,9]. Emerging surgical techniques include, first, intraocular vision-improving devices such as implantable miniature telescopes and add-on intraocular lens implants; second, cell therapy, particularly RPE transplantation, has emerged as a promising strategy for vision restoration. The first option offers advantages regarding head motion, vestibular ocular reflex development, and depth perception, whereas the second promises an ideal strategy to reconstruct the degenerated retina structure. However, all of these approaches require expensive, sophisticated tools and still present several limitations [10,11].

Beyond the lack of vision-improving treatments, GA is known to exert a significant influence on vision-related quality of life (QoL) [12]. Difficulties with reading, vision in dim illumination, face recognition, locating signs, and driving typically worsen over a two-year period in affected patients. A recent global survey further highlighted the substantial burden of GA on patients’ daily lives, emotional well-being, and independence [13,14], providing valuable insights into the unique challenges they face and underscoring the need for tailored support, new visual rehabilitation methods and educational resources.

Microperimetry (MP) is one of the non-invasive and rapid visual function tests available, and has been proposed as a useful method for detecting central retinal damage and localizing the preferred retinal locus (PRL) used for visual tasks. Five commercially available MP testing devices are currently used in research and clinical settings: MP1, MP3, Optos, Compass, and Macular Integrity Assessment (MAIA). Among these, MAIA is FDA-approved and provides fixation indices. In addition, it allows stimulation of a new, previously chosen retinal locus with greater sensitivity than the original PRL, a process known as biofeedback fixation training (BFT). BFT, a relatively recent technique grounded in neuroplasticity, has been clinically established as a promising visual rehabilitation approach for patients with central vision loss.

The aim of this study was to evaluate the effects of BFT on visual acuity and QoL in patients with GA.

## 2. Materials and Methods

### 2.1. Study Design

This prospective case-series study was conducted at the Department of Ophthalmology, Semmelweis University, Budapest, Hungary, between 2023 and 2025. The study protocol was approved by the Institutional Ethics Review Board at Semmelweis University Hungary (SE RKEB: 136/2023, SE TUKEB 248/2016, 037871/2016/OTIG, OGYÉI/42821/2019, OGYÉI/12949/2023) and adhered to the tenets of the Declaration of Helsinki. Signed informed consent was obtained from all participants after a detailed explanation of the study procedures.

### 2.2. Participants

Participants in this study were selected from among patients with GA attending the general and retina outpatient clinics. The diagnosis of dry AMD was confirmed using fundus biomicroscopy, spectral domain optical coherence tomography (SD-OCT), and OCT angiography (OCTA). Inclusion criteria were: total loss of central visual acuity or reading ability in one eye, a retinal area with good retinal sensitivity located within 20 degrees from the foveal center (serving as possible future PRL for BFT) on the other eye, with refractive error within ±5 diopters in both eyes, absence of media opacities (either natural lens or intraocular lens implantation), no history of glaucoma, macular dystrophy, previous peripheral retinal disease (e.g., diabetic retinopathy, retinal detachment), or optic nerve disease in either eye, and the ability to understand and follow instructions throughout the training.

### 2.3. Visual Acuity Measurements

A masked optometrist assessed monocular best corrected distance visual acuity (BCVA) after formal refraction using a high-contrast ETDRS chart at 4 m. BCVA for each eye was recorded as the LogMAR equivalent of the decimal score corresponding to the ETDRS rows correctly read by the patient.

Near visual acuity was measured in each eye in the best corrected state using the Radner reading chart, a standardized, multilingual, and internationally accepted method for evaluating reading performance. The chart consists of 32 sentences constructed according to linguistic statistics, considering word number, length, position, lexical difficulty, and syntactic complexity. The geometric proportions of the chart are constant, so the only variable is font size, which increases incrementally by 0.1 LogRAD units [15]. The patient’s baseline value was defined as the last line read correctly and without omissions.

Contrast sensitivity (CS) was assessed using the SpotChecks Contrast Sensitivity Test, which quantifies the ability to distinguish differences in luminance and is particularly sensitive to subtle visual changes in GA patients. The test consists of a worksheet with 24 rows and 5 columns, each cell containing a gray spot with a diameter of 9 mm, with intensity decreasing by 0.01 LogCS from top to bottom and left to right. For each patient, the total number of correctly perceived spots at best corrected vision was recorded up to the first row in which no spots were seen [16].

### 2.4. National Eye Institute Visual Functioning Questionnaire-25 (NEI-VFQ-25)

Vision-related quality of life was evaluated using the National Eye Institute Visual Function Questionnaire-25 (NEI-VFQ-25), which measures the impact of vision loss on daily activities and social functioning. The questionnaire includes subscales for general health, near and distance vision, driving, peripheral vision, color vision, eye pain, role limitations, independence, social functioning, and mental health. Each subscale is scored from 0 (worst) to 100 (best) [17]. To ensure all participants could respond, the ophthalmologist administering BFT read the questions and possible answers aloud, repeating them as needed, and the patient verbally indicated the most appropriate answer, which was recorded. Responses were scored for each subscale according to standardized procedures, and a weighted, aggregate score was calculated to quantify overall vision-related quality of life.

### 2.5. Microperimetry and Biofeedback Fixation Training

Microperimetry was performed using the MAIA (Macular Integrity Assessment) system. This device captures retinal images using Scanning Laser Ophthalmoscope (SLO) technology and projects variable intensity light stimuli onto a 10-degree diameter area at 37 predefined points to determine retinal sensitivity. Stimuli are presented as white light of decreasing intensity; the intensity increases by 4 dB until the patient indicates perception using a handheld button, then decreases in 2 dB steps until the stimulus is no longer perceived. A negative control point corresponding to the center of the blind spot is also included. Stimuli are presented in parallel in random order. During microperimetry, the MAIA system tracks eye movements at 25 Hz to record the location and stability of the patient’s current preferred fixation point (PRL) by comparing each image with the baseline SLO image. Fixation stability is quantified using P1 and P2 percentages, representing the proportion of fixation points within 1 degree (P1) and 2 degrees (P2) of the PRL, and by the bivariate contour ellipse areas (BCEA63 and BCEA95), representing ellipses containing 63% and 95% of the fixation points, respectively. Microperimetry also provides quantitative data on average retinal sensitivity and the distance between the fixation point and the fovea.

Biofeedback fixation training (BFT) is a vision rehabilitation technique designed to stabilize fixation by establishing a new PRL. Prior to training, retinal sensitivity is measured with microperimetry to select a new PRL in a functionally preserved area. Suitability for training was confirmed using a preliminary microperimetric assessment. A new fixation point was manually chosen for each patient, guided by previous studies [18,19], typically located superior to the central lesion. If the patient had an existing, appropriately located PRL, it was confirmed; otherwise, the nearest point with the best possible retinal sensitivity was selected. During BFT, the selected PRL is highlighted with a white light surrounded by a 1-degree red circle. The untreated eye is covered, and the patient is instructed to focus on the white light, maintain fixation as long as possible and to refocus promptly if fixation is lost. The operator monitors the patient’s fixation on the SLO image relative to the designated PRL. Deviations trigger a monotonous auditory signal, with frequency increasing as the fixation point approaches the target, and the operator provides verbal guidance. When fixation is achieved within the 1-degree circle, the auditory signal switches to music (e.g., Mozart), and the white light becomes fully visible, providing visual and auditory feedback. At the end of each session, patients receive feedback on the percentage of time fixation was maintained within 1-degree and 2-degree diameters around the PRL.

Training consisted of one 10 min session per week for 10 weeks. Follow-up assessments were performed at one week, one month, 6 months, and 12 months after completion of the last training session.

### 2.6. Statistical Analysis

Patients’ demographic and ocular characteristics were assessed for normality using the Shapiro–Wilk W test and are presented as counts with percentages or as means ± standard deviations, as appropriate. Continuous variables parameters were not normally distributed; therefore, non-parametric tests were applied, using appropriate rank-based methods for univariable and multivariable analyses. Statistical significance was set at *p* < 0.05. All analyses were performed using SPSS Statistics 23.0 (IBM Corp., Armonk, NY, USA).

## 3. Results

### 3.1. Demographic and Visual Performances Prior to Training

Data from 18 eyes of 18 patients (13 females, 5 males) were analyzed. The mean age was 74.0 ± 5.81 years. For nine eyes, BFT was performed as a single session consisting of 10 consecutive weekly 10 min trainings, while the other nine eyes underwent a second training session after 6 months. The mean follow-up time was 13.4 (range 3–26.8) months. Among the patients, 75.6% had comorbidities, including hypertension (50%) and diabetes mellitus (30%). None of the patients had neurodegenerative diseases, and none had experienced an accident in the 3 months preceding training. In seven cases, cataract surgery with artificial posterior chamber lens implantation had been performed more than 3 months prior to BFT.

Baseline visual function of the trained eyes included a mean BCVA of 0.7 LogMAR (SD ± 0.27), near visual acuity of 0.36 LogRAD (SD ± 0.31), and CS of 53.6 pcs (SD ± 16.7). Microperimetry revealed a mean overall retinal sensitivity of 12.1 dB (SD ± 7.3) across 37 loci. In general, 96.3% of eyes exhibited poor fixation stability. Analysis of fixation stability parameters indicated that 100% had abnormal BCEA values, 96.3% had abnormal P1, and 85.2% had abnormal P2. PRL locations were distributed as follows: superotemporal 44.4%, inferotemporal 27.8%, superonasal 22.2%, and inferonasal 5.6% (one patient), with a mean eccentric distance of 3.49 degrees (SD ± 2.58 degrees). Baseline quality of life assessments indicated that patients’ primary complaints involved reading, vision in dim light, face recognition, locating signs, and driving. Baseline visual characteristics are summarized in Table 1.

### 3.2. Effects of Biofeedback Fixation Training on Visual Performance in GA

**Figure 1:**
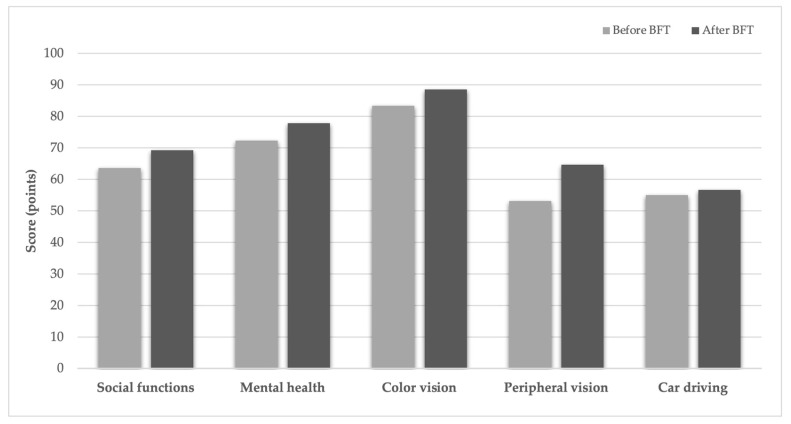
Quality-of-life changes following MAIA-based training. Scores for frequently reported symptoms across five vision-specific tasks, shown before and after BFT at the end of the follow-up period.

**Figure 2:**
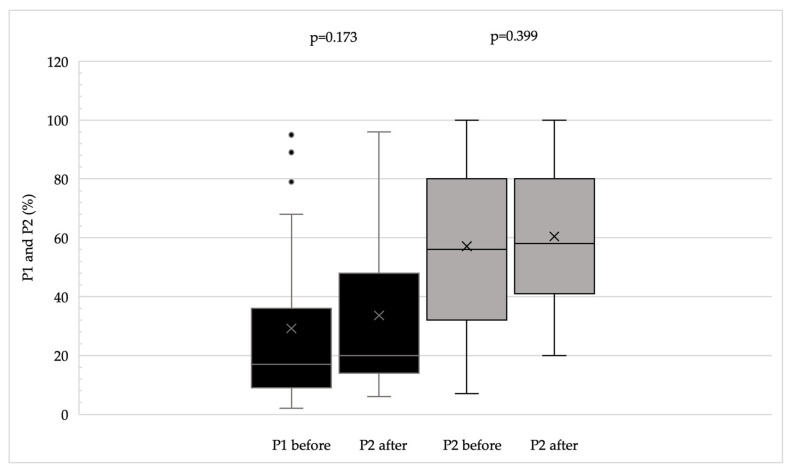
P1 and P2 values before and after BFT (at the end of the follow-up period). P1 represents the percentage of all fixation points falling within a 1-degree diameter circle, while P2 represents the percentage of all fixation points falling within a 2-degree diameter circle. Boxplots display the median (horizontal line), interquartile range (box), and minimum–maximum values excluding outliers (whiskers). The mean is indicated by an “X”. Dots represent outliers, defined as values lying more than 1.5 times the interquartile range from the quartiles.

**Figure 3:**
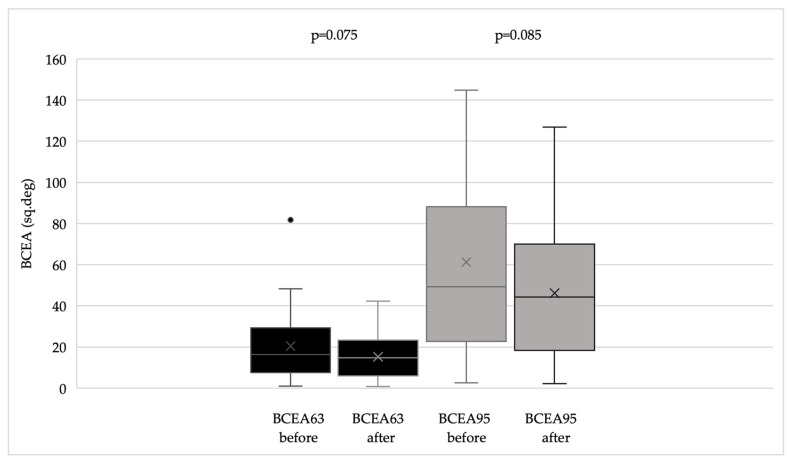
BCEA63 and BCEA95 values before and after BFT (at the end of the follow-up period). BCEA: bivariate contour ellipse area. BCEA63 represents ellipses containing 63% of the fixation points. BCEA95 represents ellipses containing 95% of the fixation points.

**Figure 4:**
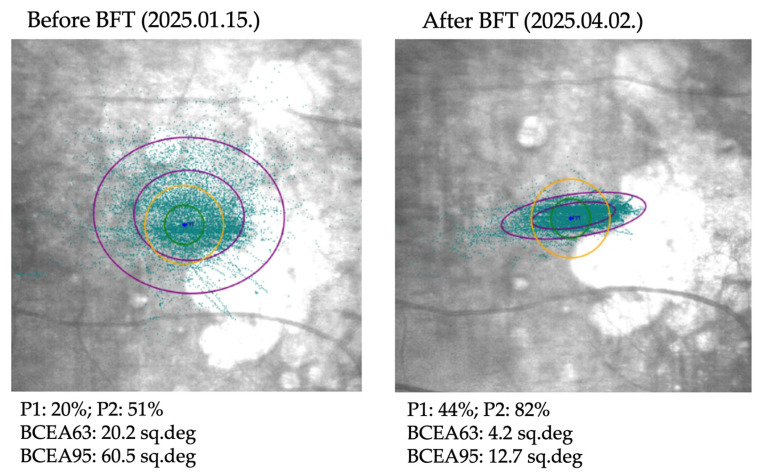
Infrared fundus images and MAIA parameters of a typical GA eye from our study cohort. The images show the left eye of a 71-year-old female patient before and after BFT. Initial visual acuity was 0.72 LogMAR, improving to 0.66 LogMAR after training. Contrast sensitivity increased by 11 units, and NEI-VFQ-25 score improved by 22 points. BCEA: bivariate contour ellipse area. BCEA63 represents ellipses (inner purple ellipses) containing 63% of the fixation points (light blue dots). BCEA95 represents ellipses (outer purple ellipses) containing 95% of the fixation points. P1 (green circle) representing the proportion of fixation points within 1 degree of the PRL (central dark blue dot). P2 (yellow circle) representing the proportion of fixation points within 2 degrees of the PRL.

BCVA improved significantly (*p* < 0.002), and both CS and reading ability (Radner test) also showed significant improvement (*p* < 0.02 for both parameters). Questionnaire scores and fixation stability indices demonstrated an overall improving trend, reflected by increased P1 and P2 values and decreased BCEA values (Figure 1, Figure 2 and Figure 3). Figure 4 illustrates the changes in fixation parameters of a representative study eye from our cohort as measured by the MAIA system before and after BFT.

In patients undergoing two BFT sessions spaced six months apart, improvements achieved after the first session were maintained, with further significant gains in vision and contrast sensitivity observed following the second session. Multivariable analysis indicated no significant difference in efficacy between the two training periods.

Radner test results were significant predictors of the composite score both before (*p* < 0.001) and after training (*p* < 0.001). In contrast, BCVA and fixation stability parameters (P1, P2, BCEA63, BCEA95) did not significantly predict the composite score either pre- or post-training (*p* > 0.05). CS significantly predicted the composite score after training (*p* = 0.003) but was not a significant predictor prior to training.

According to the multivariable model, post-training BCVA was significantly predicted by pre-training BCVA (beta = 0.515, *p* < 0.001) and age (beta = −0.116, *p* = 0.007), indicating better outcomes in younger patients with higher baseline visual acuity. Post-training reading ability was also significantly influenced by age (beta = −0.332, *p* = 0.03). Neither composite scores nor CS were significantly predicted by any examined variables. Initial fixation stability did not significantly influence final BCVA, reading ability, CS, or composite scores. Notably, age consistently emerged as a key predictor across all multivariable models, whereas initial fixation stability had no significant effect on any evaluated outcome measure.

## 4. Discussion

Our results demonstrating significant improvements in visual acuity, contrast sensitivity, and reading ability in GA eyes are consistent with previous reports on the use of BFT in AMD patients [19,20,21,22,23]. BFT has been primarily applied in central vision disorders, but promising functional outcomes have also been reported in myopia, inherited retinal degenerations, and nystagmus [19]. Despite these findings, its adoption in clinical practice remains limited due to incomplete familiarity with the procedure and inconsistent standards.

In a preliminary pilot study, our group evaluated whether BFT using the MAIA microperimeter could enhance visual performance in AMD patients. Six AMD patients (a mixed cohort of nAMD and GA eyes) underwent twelve 10 min biofeedback sessions over approximately three months, while five age-matched healthy participants served as untrained controls. The AMD group showed significant improvements in spatial luminance contrast sensitivity, particularly at low spatial frequencies, suggesting enhanced processing of coarse visual information. Although visual acuity, color discrimination, and reading speed did not change markedly, participants reported a noticeable subjective improvement in perceived visual quality [24,25].

Based on these favorable findings, we focused specifically on patients with GA, who experience progressive retinal atrophy and commonly develop central and/or paracentral scotomas [12,13]. Patients with central vision loss often utilize a larger retinal area for fixation when asked to fixate eccentrically, but this fixation instability reduces reading speed and can impair more complex tasks such as face recognition, as also observed in other disorders, including social phobias, Williams syndrome, autism, schizophrenia, and prosopagnosia [12,13].

Macular disease patients adopt self-compensatory strategies by using peripheral retinal areas known as preferred retinal loci (PRL). An optimal PRL maintains visual images on a discrete and stable retinal area (fixation stability), supports pursuit of moving targets, and allows rapid saccadic shifts to other objects. While many patients use PRLs in healthy peripheral macula, these locations are not always ideal, resulting in suboptimal fixation stability [24,26]. In our cohort, even the better eye, particularly the study eye, demonstrated poor fixation stability and an existing extrafoveal PRL, most frequently superotemporal. This distribution aligns with literature, where the majority of PRLs are located in the upper and right quadrants of the retina, corresponding to the inferior and left visual field, which supports ocular locomotion and reading performance. However, PRL function does not depend solely on its position relative to the scotomas [26,27,28]. Riss-Jayle et al. also showed that when a PRL is placed 20 degrees from the foveal center, it loses effectiveness because it no longer meets the necessary oculomotor criteria [29]. In our study, PRL eccentricity was under 5 degrees, likely contributing to training success. Following previously established guidelines, if patients had a suitably located PRL, it was confirmed; otherwise, the closest point with optimal retinal sensitivity was selected.

Several studies have investigated the potential benefits of rehabilitation strategies in patients with AMD, employing various approaches. Initially, acoustic biofeedback was hypothesized to assist the central nervous system in establishing a newly developed PRL by modulating attention, thereby training patients to use the most functionally advantageous retinal area to optimize residual vision. Subsequently, acoustic biofeedback was combined with a visual light stimulus to enhance both fixation stability and PRL localization, yielding superior outcomes in fixation stability, reading speed, and visual acuity compared with acoustic feedback alone [23]. The superiority of this combined approach was further supported by Amore et al., who demonstrated improvements in retinal sensitivity, suggesting that intermittent receptor activation can transmit increased macular input to the visual cortex [19]. In our cohort, we applied the same combined strategy, although retinal sensitivity did not significantly improve.

Consistent with previous studies, training consisted of 10 min weekly sessions on a single eye for 10 consecutive weeks [19,20,21,22,23]. Following BFT, patients reported not only objective improvements in reading ability and visual acuity but also subjective enhancements in daily activities [19,20,21,22,23]. Data on the duration of these beneficial effects remain limited. In our cohort, the results of the first BFT period were maintained with a second session six months later, with multivariable analysis showing no significant difference in efficacy between sessions. The observed increases in NEI-VFQ-25 scores for frequent symptoms across five vision-specific tasks further support the long-term sustainability of BFT effects.

Our results suggest that CS and reading ability are the primary determinants of Qol in patients with GA, rather than initial fixation stability, as they significantly predicted BCVA and NEI-VFQ-25 composite scores following BFT. Age was also a key factor, with younger patients with better baseline BCVA achieving superior outcomes. The rationale for BFT in macular disease is grounded in neuroplasticity, which contributes to the reorganization of the primary visual cortex following central vision loss and eccentric PRL training. Strong activation of the visual cortex has been observed in patients with central visual loss during fixation using eccentric PRLs [26]. While brain plasticity has traditionally been considered maximal in youth and declining with age, recent evidence indicates that neuroplasticity persists throughout life, supported by advances in neuroimaging of brain structure and function [30]. Although skill acquisition may be slower in older populations, our findings on the robust role of age in long-term BFT outcomes underscore the importance of tailoring rehabilitative protocols to specific macular diseases.

This study has several limitations. The small sample size precluded detailed sub-analyses of BFT efficacy or correlations with other measured and epidemiological parameters. Additionally, heterogeneity and lack of standardization in BFT protocols in the literature pose challenges. Strengths of the study include the relatively long follow-up, a homogeneous patient cohort, and the use of extensive assessments to obtain a comprehensive evaluation of Qol and daily activities in patients with GA.

## 5. Conclusions

BFT appears to be a durable and effective tool for visual rehabilitation in patients with GA, as it facilitates long-term retention of an efficient PRL through enhanced attentional modulation. Given the substantial burden of GA, patients represent ideal candidates for this rehabilitation approach, as the disease remains incurable and progressive central vision loss significantly impairs Qol, independence, and emotional well-being. Our results suggest that optimal outcomes are achieved when BFT is initiated in the better-seeing eye soon after central vision loss in the fellow eye, in younger patients, and in those with higher baseline monocular BCVA. Looking forward, in combination with emerging therapies that slow or halt disease progression, BFT could serve as a valuable adjunctive intervention to help elderly patients maintain independence and an active lifestyle.

## Figures and Tables

**Table 1:** Baseline visual characteristics and fixation stability in our patient cohort.

Parameter	Value
BCVA (LogMAR)	0.70 ± 0.27
Radner test (LogRAD)	0.36 ± 0.31
SpotChecks test (pcs)	53.59 ± 16.72
NEI-VFQ-25 composite score mean ± SD	61.24 ± 14.61
P1 mean ± SD and abnormal (<95%) (*n*%)	29.22 ± 26.17 (96.3%)
P2 mean ± SD and abnormal (<95%) (*n*%)	57.11 ± 27.69 (85.2%)
PRL distance mean ± SD (deg)	3.49 ± 2.58
BCEA63 (sq.deg) mean ± SD, abnormal (<0.8 sq.deg) (*n*%)	20.44 ± 18.79 (100%)
BCEA95 (sq.deg) mean ± SD, abnormal (<2.4 sq.deg) (*n*%)	61.25 ± 55.32 (100%)

BCVA: best corrected visual acuity; P1: percentage of all fixation points falling within a 1-degree diameter circle; P2: percentage of all fixation points falling within a 2-degree diameter circle; PRL: preferred retinal loci; BCEA: bivariate contour ellipse area.

## Data Availability

Datasets are deposited in Dryad database (https://doi.org/10.5061/dryad.ngf1vhj81).

## Abbreviations

The following abbreviations are used in this manuscript:

AMD	age-related macular degeneration
BCEA63	bivariate contour ellipse area 63
BCEA95	bivariate contour ellipse area 95
BCVA	best corrected visual acuity
BFT	biofeedback fixation training
CS	contrast sensitivity
ETDRS	Early Treatment Diabetic Retinopathy Study
FDA	Food and Drug Administration
GA	geographic atrophy
MAIA	Macular Integrity Assessment System
MP	microperimetry
nAMD	neovascular age-related macular degeneration
NEI-VFQ-25	Visual Functioning Questionnaire-25
OCT	optical coherence tomography
OCTA	optical coherence tomography angiography
PRL	preferred fixation locus
QoL	quality of life
SD-OCT	spectral domain optical coherence tomography
SLO	Scanning Laser Ophthalmoscope
